# Optimal duration of dual antiplatelet therapy for coronary artery disease

**DOI:** 10.1007/s12471-018-1113-5

**Published:** 2018-04-30

**Authors:** W. J. Kikkert, P. Damman

**Affiliations:** Academic Medical Center, Department of Cardiology, University of Amsterdam, F3-155, Amsterdam, The Netherlands

**Keywords:** DAPT, Short-term, Long-term

## Abstract

The optimal duration of dual antiplatelet therapy (DAPT) for stable coronary artery disease and acute coronary syndrome is a complex decision. We review current literature on standard duration DAPT versus short duration DAPT (6 months or shorter) or extended duration DAPT (>12 months) after percutaneous coronary intervention with drug-eluting stent placement, and prolonged treatment after 12 months in acute coronary syndrome. Current guideline recommendations are summarised, including the use of risk scores for ischaemic and bleeding risk assessment. Because of the limitations of current risk scores, we propose multiple patient-related and procedure-related factors for the ischaemic and bleeding risk assessment aiding in personalised DAPT duration.

## Introduction

Dual antiplatelet therapy (DAPT) is directed at preventing stent thrombosis, myocardial infarction, and stroke and it is standard therapy after percutaneous coronary intervention (PCI) and after acute coronary syndrome (ACS). In the Netherlands more than 45,000 patients are treated with percutaneous coronary intervention each year. In Europe, it is estimated that approximately 1,400,000 and 2,200,000 patients per year may have an indication for DAPT after percutaneous coronary intervention or myocardial infarction, respectively [1].

Colombo et al. introduced a strategy of improved bare-metal stent (BMS) expansion with high-pressure balloon inflation guided by intravascular ultrasound images first [2]. Low rates of stent thrombosis were achieved with the combination of this stent technique and DAPT consisting of aspirin and 1 month of ticlopidine, and this proved to be the advent of DAPT as we know it today.

Drug-eluting stents (DES) were subsequently introduced to mitigate the risk of restenosis. However, concerns over late and very late stent thrombosis occurring after first-generation DES implantation increased the need for long-term DAPT [3]. However, the risks of late and very late stent thrombosis have declined considerably since the introduction of second generation DES [4]. In conjunction with the results of recent randomised controlled trials (RCTs) that investigated shorter duration of DAPT, this has led to a rapid paradigm shift in the way the clinical community perceives DAPT duration.

Conversely, among patients with acute coronary syndrome, the risk of myocardial infarction outside the stented segment remains high—well beyond 12 months after the initial procedure [5]. Twenty percent of patients with myocardial infarction who are event free the first year, suffer a major adverse cardiovascular event between 1 and 4 years after their index event [6]. Moreover, among patients with ST-elevation myocardial infarction (STEMI) the average daily risk of ischaemia exceeds the average daily risk of bleeding beyond 12 months after the initial myocardial infarction [7]. Given the overwhelming evidence that long-term DAPT reduces myocardial infarction outside the stented segment as well as stroke, continued DAPT 12 months after the initial myocardial infarction might improve the net clinical outcome.

In light of the above, choosing the optimal DAPT duration has become more complex than ever. The aim of this manuscript is to provide guidance in determining the optimal DAPT duration for individual patients.

**Figure:**
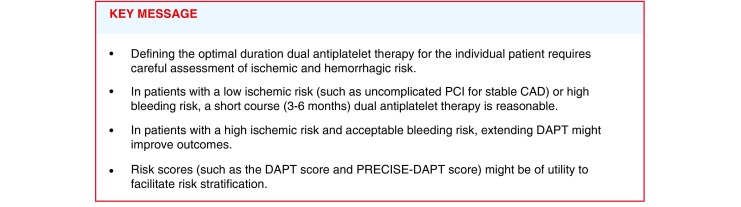

## Available evidence

### Duration of DAPT in stable coronary artery disease (CAD)

Based on results of the CHARISMA (Clopidogrel for High Atherothrombotic Risk and Ischemic Stabilization, Management, and Avoidance) study [8], DAPT is currently not indicated for medically managed patients with stable CAD and no history of myocardial infarction [9]. However, after PCI with stent implantation, DAPT is standard practice. To date, there have been no randomised comparisons of different DAPT durations in a dedicated study confined to stable CAD patients undergoing PCI. Several studies have established the 1‑month course of DAPT after bare-metal stent implantation [10–12]. Owing to the increased risk of late stent thrombosis, an arbitrary minimal 12-month DAPT duration has been subsequently recommended based on expert opinion after first generation DES, irrespective of clinical presentation. Therefore, recommendations regarding stable CAD patients undergoing PCI are based on subgroup analyses from the pivotal RCTs discussed in the following section.

### Short duration of DAPT (6 months or shorter) after PCI with DES for stable CAD or ACS

Tab. 1 provides an overview of characteristics and outcomes of studies comparing different durations DAPT after PCI with DES. Five RCTs have compared a shorter duration (3–6 months) DAPT with 12 months DAPT after PCI with DES for various indications [13–17]. The main endpoints of these non-inferiority trials were composite ischaemic events (or a composite of ischaemic and haemorrhagic events) and stent thrombosis. In these studies, 24–75% of patients presented with ACS. Nonetheless, most of these studies included a study population with a low risk of ischaemic events. For instance, in EXELLENT (Efficacy of Xience/Promus Versus Cypher to Reduce Late Loss After Stenting) and SECURITY (Second-Generation Drug-Eluting Stent Implantation Followed By Six- Versus Twelve-Month Dual Antiplatelet Therapy) only biomarker-negative patients with ACS were included [15, 17]. In OPTIMIZE (Optimized Duration of Clopidogrel Therapy Following Treatment With the Zotarolimus-Eluting Stent in Real-World Clinical Practice), STEMI patients were excluded [14]. Moreover, in the majority of these studies, patients were randomised at the time of PCI, and not at the time of DAPT discontinuation, thus including events occurring while both treatment groups were on DAPT, potentially diluting differences in ischaemic events arising after DAPT cessation at 3 or 6 months in the shorter DAPT arm. Therefore, given that these studies were non-inferiority studies, the study results should be interpreted with caution. Finally, non-inferiority margins were wide in some studies (much wider than would be deemed clinically relevant differences), inclusion of patients in some studies terminated prematurely, event rates turned out much lower than anticipated and, therefore, all of these studies were underpowered to detect differences in hard clinical endpoints such as stent thrombosis.

**Table 1:** Studies comparing different durations of dual antiplatelet therapy after PCI

Study (year)	Randomisation	Sample size	Primary endpoint	Design and randomisation	% ACS	Proportion with Newer-Generation DES (%)	Primary endpoint (short vs. long DAPT)
RESET (2012) [13]	3 vs. 12 months DAPT	2,117	Cardiac death, MI, ST, revasc. or bleeding	Non-inferiority Randomisation at time of PCI	55	85	4.7% in both arms (*p*_NI_ <0.001)
OPTIMIZE (2014) [14]	3 vs. 12 months DAPT	3,119	NACCE—death, MI, stroke, or bleed	Non-inferiority Randomisation at time of PCI	32	100	6.0% with 3 months DAPT vs. 5.8% with 1‑year DAPT (*p*_N__I_ = 0.002)
EXCELLENT (2011) [15]	6 vs. 12 months DAPT	1,443	Cardiac Death, MI, or ischemia driven TVR	Non-inferiority Randomisation at time of PCI	51	75	4.8% with 6‑months vs. 4.3% with 1‑year DAPT (*p*_NI_ = 0.001)
ISAR-SAFE (2014) [16]	6 vs. 12 months DAPT	4,000* (planned: 6,000)	Death, MI, stroke, or TIMI major bleed	Non-inferiority Randomisation at DAPT discontinuation	40	72	1.5% with 6 months DAPT vs. 1.6% with 1‑year DAPT (*p*_N__I_ < 0.001)
SECURITY (2014) [17]	6 vs. 12 months DAPT	1,399* (planned: 2,740)	Cardiac death, MI, ST, or stroke	Non-inferiority Randomisation at time of PCI	38	100	4.5% with 6‑months DAPT vs. 5.7% with 1‑year DAPT (*p*_NI_ ≤ 0.05)
PRODIGY (2012) [20]	6 vs. 24 months DAPT	1,970	Death, MI, stroke	Superiority Randomisation 1 month after PCI	75	50	10.0% with 6 months DAPT vs. 10.1% with 2‑year DAPT (*p* = 0.91)
ITALIC (2014) [21]	6 vs. 24 months DAPT	1,822* (planned: 2,475)	Death, MI, urgent TVR, stroke or bleeding	Non-inferiority Randomisation at time of PCI	24	100	1.6% with 6 months DAPT vs. 1.5% with 2‑year DAPT
ARCTIC Interruption (2014) [22]	12 vs. 18–24 months	1,259	Death/MI/ST/CVA/TVR	Superiority Randomisation at DAPT discontinuation	26	63	4.0% in both arms (median 17 months FU) (*p* = 0.58)
DAPT (2015) [23]	12 vs. 30 months	9,961	1 ST 2 MACE	Superiority Randomisation at DAPT discontinuation	43	59	ST: 1.4% vs. 0.4% and MACE 4.1 vs. 2.1% (*p* < 0.001)
DES-LATE (2010) [24]	12 vs. 36 months	5,045	Cardiac death/MI/ CVA	Superiority Randomisation at DAPT discontinuation	61	30	2.4% SAPT vs. 2.7% DAPT (*p* = 0.75)
OPTIDUAL (2015) [25]	12 vs. 48 months	1,385* (planned: 1,966)	Death/MI/ CVA/bleeding	Superiority Randomisation at DAPT discontinuation	36	59	7.5% SAPT vs. 5.8% DAPT (*p* = 0.17)

^*^ Inclusion of patients terminated prematurely

*RESET* REal Safety and Efficacy of 3‑month dual antiplatelet Therapy following Endeavor zotarolimus-eluting stent implantation, *NI* non-inferior, *OPTIMIZE* Optimized Duration of Clopidogrel Therapy Following Treatment With the Zotarolimus-Eluting Stent in Real-World Clinical Practice, *EXCELLENT* Efficacy of Xience/Promus Versus Cypher to Reduce Late Loss After Stenting, *ISAR-SAFE* Intracoronary Stenting and Antithrombotic Regimen: Safety And EFficacy of 6 Months Dual Antiplatelet Therapy After Drug-Eluting Stenting, *SECURITY* Second-Generation Drug-Eluting Stent Implantation Followed By Six- Versus Twelve-Month Dual Antiplatelet Therapy, *PRODIGY* Prolonging Dual Antiplatelet Treatment After Grading Stent-Induced Intimal Hyperplasia Study, *ITALIC* Is There a Life for DES After Discontinuation of Clopidogrel, *ARCTIC* Assessment with a double Randomization of (1) a fixed dose versus a monitoring-guided dose of aspirin and Clopidogrel after DES implantation, and (2) Treatment Interruption versus Continuation, 1 year after stenting, *DAPT* Dual AntiPlatelet Therapy, *DES-LATE* Optimal Duration of Clopidogrel Therapy With DES to Reduce Late Coronary Arterial Thrombotic Event, *OPTIDUAL* OPTImal DUAL antiplatelet therapy, *CVA* cerebrovascular accident, *DAPT* dual antiplatelet therapy, *DES* drug-eluting stent, *MACE* major adverse cardiac event, *MI* myocardial infarction, *NACCE* Net Adverse clinical and cerebral event,* PCI* percutaneous coronary intervention, *SAPT* single antiplatelet therapy,* ST* stent thrombosis. *TVR* target vessel revascularisation

Nonetheless, none of these studies or subsequent meta-analyses found an increased risk of stent thrombosis or major adverse cardiac events (MACE) with a shorter duration of DAPT. On the contrary, short-duration DAPT was associated with fewer bleeding complications [18, 19]. Taken together, these studies demonstrate that in patients with a low ischaemic risk or with a high bleeding risk, a shorter duration of DAPT may be reasonable in patients after uncomplicated elective PCI with second generation DES (e. g., everolimus-eluting or zotarolimus-eluting), or patients with ACS at relatively high bleeding risk.

### Extended duration of DAPT (>12 months) after PCI with DES for stable CAD or ACS

Six RCTs, consisting predominantly of patients treated with elective DES implantation, compared prolonged DAPT (total therapy duration: 18–48 months) with 6–12 months of DAPT (Tab. 1; [20–25]). Four of these studies were underpowered to detect differences in major endpoints. In ITALIC (Is There a Life for DES After Discontinuation of Clopidogrel) patients were randomised at the time of PCI and in PRODIGY (Prolonging Dual Antiplatelet Treatment After Grading Stent-Induced Intimal Hyperplasia Study) patients were randomised one month after PCI, potentially diluting differences in outcome between the two study groups [20, 21].

In the DAPT (Dual Antiplatelet Therapy) study, patients who did not suffer a major adverse cardiac event or bleeding in the first year following DES implantation, were randomised to an additional 18 months of DAPT or to aspirin monotherapy. Extended DAPT was associated with a 0.7% absolute reduction in stent thrombosis, a 1.6% absolute reduction in MACE, and a 0.9% absolute increase in GUSTO (global use of strategies to open occluded coronary arteries) moderate or severe bleeding [26]. Unexpectedly, there was a borderline significant increase in overall mortality (0.5% absolute increase) with prolonged DAPT, which was the result of a statistically significant increase in non-cardiovascular mortality, mainly related to increased cancer-related mortality [27]. This may simply reflect a play of chance: cancer had been diagnosed prior to enrolment in nine patients who subsequently died, eight of whom were randomly allocated to extended DAPT [27].

The meta-analyses conducted after publication of the DAPT study, demonstrated a reduction in stent thrombosis and myocardial infarction with extended DAPT, but this did not translate into a reduction in cardiovascular mortality. In fact, several of these meta-analyses found an increased risk of non-cardiovascular and all-cause mortality with extended DAPT after PCI [18, 19, 28]. These meta-analyses, however, did not include the OPTIDUAL (OPTImal DUAL antiplatelet therapy) study. Moreover, none of the 11 RCTs conducted so far were powered for mortality. In the systematic review and meta-analysis conducted by the Evidence Review Committee (ERC) of the American College of Cardiology/American Heart Association Task Force on Clinical Practice Guidelines, including the OPTIDUAL study, no statistically significant difference in mortality was observed between extended DAPT and short-term DAPT [29]. However, when trials were stratified into completed trials or incomplete trials with slow enrolment, there was weak evidence for increased mortality. A subanalysis from the DAPT trial shows that this might be explained by patients with stable CAD [30]. In summary, whether extended DAPT is associated with excess mortality in patients treated with PCI remains uncertain and deserves further research.

### Duration of dual antiplatelet therapy in ACS

The currently recommended duration of DAPT for patients with ACS, non-ST-segment elevation ACS (NSTE-ACS) as well as ST-segment elevation ACS (STE-ACS) is 12 months [9]. This recommendation is based on the CURE (Clopidogrel in Unstable Angina to Prevent Recurrent Events) trial, the PLATO (Platelet Inhibition and Patient Outcomes) study and the TRITON-TIMI 38 (Trial to Assess Improvement in Therapeutic Outcomes by Optimizing Platelet Inhibition with Prasugrel—Thrombolysis in Myocardial Infarction 38). In the CURE trial, patients with NSTE-ACS were randomised to clopidogrel for 3–12 months (median 9 months) or placebo, in addition to aspirin. The majority of patients were treated without revascularisation, though a reduction in ischaemic events was observed both in those treated with revascularisation (PCI or coronary artery bypass grafting) and in those treated with medical therapy alone [31]. In PLATO, treatment with ticagrelor during an intended 12 months (but actual median 9 months) was associated with a reduction in the primary endpoint, cardiovascular death, myocardial infarction, or stroke, as compared with clopidogrel in patients with NSTE-ACS and STEMI [32]. In TRITON-TIMI, 38 prasugrel for a median duration of 14.5 months reduced the primary endpoint cardiovascular death, myocardial infarction, or stroke, as compared with clopidogrel in ACS patients undergoing PCI in the setting of ACS [33].

The results of the CURE trial have been extrapolated to patients with STEMI on the basis of the fact that in both NSTE-ACS and STEMI coronary thrombosis is the final instigating event leading to ischaemia. Based on this consideration, as well as on the results from the PLATO and TRITON-TIMI 38 trials, it is recommended that patients with STEMI are treated with DAPT for 12 months [9].

A shorter duration of DAPT after ACS was investigated in the studies discussed under section “*Short duration of DAPT (6 months or shorter) after PCI with DES for stable CAD or ACS”. *These studies were, however, not dedicated ACS studies, and most studies only included low-risk ACS.

In CHANGE DAPT, a single centre, observational study comparing clopidogrel with ticagrelor in ACS, ticagrelor was not associated with better outcomes as compared with clopidogrel. The study was conducted at Thoraxcentrum Twente, where on 1 May 2014 the use of clopidogrel was replaced by ticagrelor. The investigators compared 1,053 patients treated with ticagrelor with a historical control group (*n* = 1,009) treated with clopidogrel before 1 May 2014. Ticagrelor was not associated with a reduction in MACE, but it was associated with an increase in major bleeding [34].

### Extended DAPT after history of myocardial infarction

In the PEGASUS-TIMI 54 (Prevention of Cardiovascular Events in Patients with Prior Heart Attack Using Ticagrelor Compared to Placebo on a Background of Aspirin-Thrombolysis in Myocardial Infarction 54) trial, 21,162 patients with a history of MI and at least one high-risk feature were randomised to ticagrelor at a dose of 90 mg twice daily, ticagrelor at a dose of 60 mg twice daily, or placebo on top of aspirin. After a median of 33 months, both doses of ticagrelor significantly reduced the risk of cardiovascular death, myocardial infarction, or stroke and increased the risk of major bleeding. The lowest number to treat and highest number to harm was observed with the dose of 60 mg twice daily [35]. A subanalysis showed that patients with lower extremity arterial disease, known to be at greater ischaemic risk, had a heightened benefit from extended ticagrelor [36]. A meta-analysis including PEGASUS-TIMI 54 and various subanalyses showed a reduction in cardiovascular death, myocardial infarction, and stroke with extended DAPT, at the cost of increased major bleeding [37].

### DAPT duration after PCI for complex lesions

Recently 2 studies have been published that investigated DAPT duration after complex PCI. These studies showed that complex PCI was an independent predictor of ischaemic events in the first year, but not of ischaemic events beyond 12 months after PCI. In an individual patient pooled meta-analysis consisting of studies comparing 3–6 months DAPT with 12–24 months DAPT, 12–24 months DAPT was associated with significant reductions in MACE compared with 3–6 months DAPT [38]. Conversely, in the DAPT study among patients without events in the first 12 months, the benefits of extending DAPT beyond one year were similar in subjects with and without complex lesions [39]. These findings suggest that complex PCI may be a more useful discriminator for predicting the benefit of DAPT within the first year after PCI, but less useful for predicting benefit of longer durations after 12 months. However, we have to keep in mind that the DAPT trial included relatively low-risk patients, and some high-risk lesions (stent placement with stent diameter <2.25 mm or >4.0 mm) were excluded.

## The European Society of Cardiology (ESC) 2017 focused update on DAPT

Currently available studies on DAPT duration are somewhat confusing. On the one hand, studies investigating a short course DAPT established non-inferiority of 3–6 months DAPT as compared with 12 months DAPT. On the other hand, pivotal trials such as the DAPT study and PEGASUS-TIMI 54 study, showed the superiority of extended DAPT over 12 months DAPT in terms of a reduction in ischaemic events. How can these contradicting findings be explained? First, the aforementioned weaknesses in study design (randomisation at the time of PCI—not at DAPT discontinuation, lower than anticipated event rates, wide pre-specified non-inferiority margins) might have contributed to type II error in the short-term DAPT trials (e. g. erroneously concluding that short-term DAPT is non-inferior to standard DAPT). Second, short-term DAPT trials included patients with a low ischaemic risk, whereas superiority of long term DAPT was predominantly shown in patients with a high ischaemic risk. This indicates that a short-term DAPT course might be non-inferior in low-risk patients, but in high-risk patients extended DAPT might be beneficial. In line with this interpretation, the recommended duration of DAPT in the recently published ESC 2017 focused update on DAPT is dependent on the indication and the thrombotic and bleeding risk [9]. In patients with stable CAD undergoing PCI with DES, DAPT with aspirin and clopidogrel is recommended for 6 months [9]. Shorter DAPT for 3 months, might be considered in patients at high bleeding risk (or 1 month in patients with extremely high bleeding risk, such as those undergoing non-deferrable surgery with high bleeding risk). In patients who have tolerated DAPT without a bleeding complication and who are at low bleeding risk but high thrombotic risk, continuation of DAPT with clopidogrel for 6–30 months may be considered.

With regards to ACS patients, DAPT consisting of a P2Y12 inhibitor on top of aspirin is recommended for 12 months. In patients who have a high bleeding risk, discontinuation of P2Y12 inhibitor therapy after 6 months should be considered. Continuation of DAPT for longer than 12 months may be considered if it is tolerated without any bleeding complications [9].

### Alternative antithrombotic therapies for secondary prevention

Since the introduction of the ESC focused update on DAPT, several important trials have been published expanding the antithrombotic therapy options for secondary prevention in coronary artery disease. With regards to prolonged antithrombotic treatment in patients with stable angina, the COMPASS trial evaluated whether rivaroxaban alone or in combination with aspirin would be more effective than aspirin alone for secondary cardiovascular prevention. Around 27,000 patients with stable atherosclerotic disease were included and followed for a mean duration of two years. Patients who were assigned to rivaroxaban (2.5 mg twice daily dose) plus aspirin had better cardiovascular outcomes and more major bleeding events than those assigned to aspirin alone. Rivaroxaban (5 mg twice daily) alone did not result in better cardiovascular outcomes than aspirin alone and resulted in more major bleeding events [40]. In patients with an indication for prolonged intensive antithrombotic therapy, the specific combination therapy that may be best for reducing the risk of major thrombotic events is unclear. No data are currently available comparing prolonged DAPT with the aspirin plus rivaroxaban combination, and differences in study populations limit a direct comparison of available evidence.

An alternative treatment option to shorter DAPT in ACS patients who have a high bleeding risk has been investigated in the TROPICAL-ACS trial. In this trial, ACS patients who underwent guided PCI were randomised to de-escalation of antiplatelet treatment or standard treatment. Because the greatest anti-ischaemic benefit of potent antiplatelet drugs over the less potent clopidogrel occurs early, while most excess bleeding events arise during chronic treatment, the investigators introduced a de-escalation regimen of DAPT involving switching from prasugrel to clopidogrel after one week. This differentiated way of treating ACS patients with clopidogrel might reduce bleeding risks compared with extended treatment with a more potent antiplatelet agent such as prasugrel. However, guided de-escalation of antiplatelet treatment was non-inferior to standard treatment with prasugrel at 1 year after PCI in terms of net clinical benefit, and bleeding outcomes were comparable [41].

Both studies investigated new treatment options, and the major challenge for clinicians, regulatory agencies, guideline committees and further studies will be identifying the patients who will benefit from these novel treatment strategies.

## Risk stratification

Taken as a whole, current evidence shows that extended DAPT is associated with a reduction in ischaemic risk that is similar in magnitude to the increase in haemorrhagic risk. In an analysis by the ERC, among DES-treated patients, extended DAPT was associated with 6 fewer myocardial infarctions and 3 fewer stent thromboses but 5 additional major bleeding events per 1,000 patients treated with DAPT per year [29]. Likewise, the PEGASUS-TIMI 54 study showed that for every 1,000 patients treated with ticagrelor 60 mg twice daily, there were 4 fewer ischaemic events but 3 more major bleeding events per year. Thus, it is clear that not all patients benefit from prolonged DAPT; patients who have a high risk of ischaemic events and a relatively low risk of bleeding events are likely to benefit from prolonged DAPT. To facilitate risk stratification in order to maximise ischaemic protection and minimise bleeding risks in the individual patient risk scores could be useful. The ESC focused update on DAPT currently states that the use of risk scores may be considered to evaluate the benefits and risks of different DAPT durations (Class IIB recommendation) [9].

The DAPT score is a combined ischaemic and haemorrhagic risk score ranging from −2 (high bleeding risk, low ischaemic risk) to 10 points (high ischaemic risk, low bleeding risk), including nine risk factors (age, congestive heart failure/low left ventricular ejection fraction (LVEF), vein graft stenting, myocardial infarction at presentation, prior myocardial infarction or PCI, diabetes, stent diameter <3 mm, smoking and paclitaxel-eluting stent placement) [42]. The DAPT score was developed in 11,648 patients who were enrolled in the DAPT study and it was externally validated in 8,136 patients enrolled in the PROTECT (Patient-Related Outcomes With Endeavor vs. Cypher Stenting) trial. It had modest predictive ability for both ischaemic and bleeding events (c-statistic 0.70 and 0.68 respectively and 0.64 and 0.64 in the PROTECT dataset), and important clinical risk factors for both bleeding (such as previous bleeding, anaemia, thrombocytopenia, and bleeding diathesis) and ischaemia (multivessel disease, stent length, bifurcation stenting) were not included in the risk score. Application of the DAPT score in the DAPT study demonstrated a reduced net adverse clinical events (combination of ischaemic and haemorrhagic events) with prolonged DAPT when compared with a standard course DAPT. Conversely, in those with a low DAPT score (<2), extended DAPT was associated with an increase in net adverse events [42]. Thus, the risk/benefit ratio of prolonged DAPT was favourable among those with a high DAPT score (≥2), e. g. low bleeding risk, high ischaemic risk. One has to keep in mind that de DAPT score was developed in a patient population in which standard versus prolonged DAPT was compared, and it might not be relevant when choosing between short and standard DAPT duration.

Recently, the PRECISE-DAPT risk score was developed and validated retrospectively in large, randomised, patient cohorts comparing the duration of DAPT [43]. The PRECISE-DAPT-score is a five-item risk score that identifies patients with a high bleeding risk after ACS, concluding that longer duration of DAPT significantly increases the bleeding risk in patients with a high PRECISE-DAPT-score. Conversely, shorter duration of DAPT in patients without a high bleeding risk increases ischaemic risk in patients with a low PRECISE-DAPT-score. The novel score was assessed within patients randomised to different DAPT durations to identify the effect on bleeding and ischaemia of a long (12–24 months) or short (3–6 months) treatment in relation to baseline bleeding risk.

## Personalised treatment and risk stratification in daily practice

Determining DAPT duration requires close consideration of clinical and procedural risk factors for ischaemic events and bleeding that are not included in the currently available risk scores. Thus, when using risk scores to guide decisions on DAPT duration, physicians should bear in mind that the risk scores are not comprehensive and that a complete analysis of patient and procedural factors is mandatory to determine DAPT duration. An important contributing factor in personalised treatment is the indication for oral anticoagulants. Extensive research is being performed on this subject, and recommendations are summarised in the latest ESC DAPT guidelines [9]. A more comprehensive review is beyond the scope of this manuscript.

**Fig. 1:**
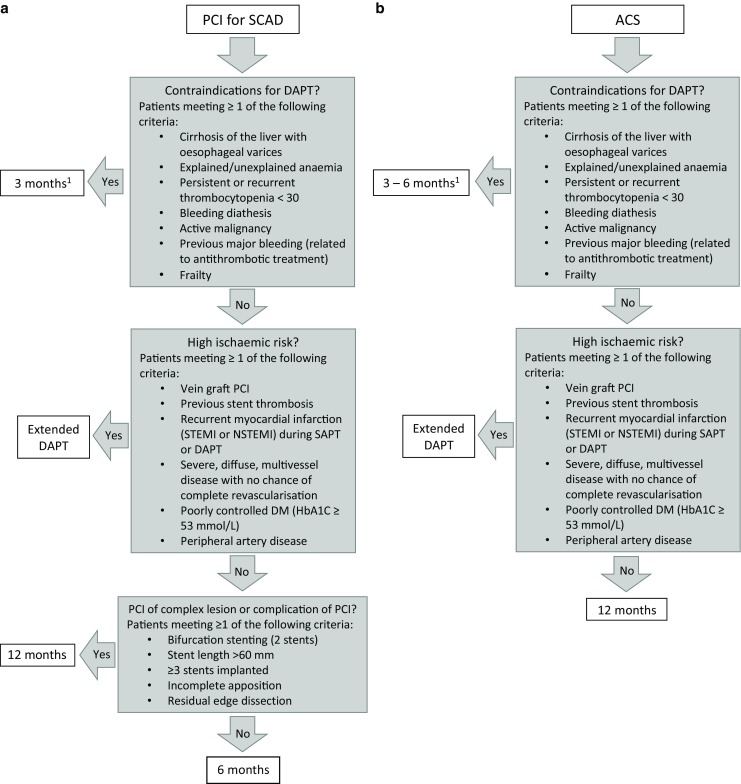
**a** Flowchart illustrating the recommended duration of dual antiplatelet therapy in patients with stable coronary artery disease according to clinical and procedural risk factors for bleeding and ischaemia. **b** Flowchart illustrating recommended duration of dual antiplatelet therapy in patients with acute coronary syndrome (^1^In patients with an extremely high bleeding risk, such as those scheduled for non-deferrable surgery with high bleeding risk, a minimum of one month of DAPT is mandatory. *ACS* acute coronary syndrome, *DM* diabetes mellitus*, DAPT* dual antiplatelet therapy, *NSTEMI* non-ST-segment elevation myocardial infarction, *PCI* percutaneous coronary intervention, *SAPT* single antiplatelet therapy*, SCAD* stable coronary disease, *STEMI* ST-segment elevation myocardial infarction)

Ultimately, the decision for whom to shorten or continue DAPT is a complex one and requires close collaboration between the interventional cardiologist and the cardiologist at the clinic or outpatient department. Based on coronary anatomy and procedural aspects such as stent length, stent apposition, and bifurcation stenting, the interventional cardiologist should provide a recommendation on DAPT duration. In the end the cardiologist who is the patient’s case manager is the one responsible for the DAPT duration considering bleeding risk, the recommendation of the interventional cardiologist, risk scores, and other, non-procedure-related, clinical risk factors, such as recurrent myocardial infarction and previous stent thrombosis. Fig. 1 shows a flowchart depicting the recommended duration of DAPT after PCI for stable CAD, and ACS, from our perspective. Current evidence supports a DAPT duration for up to 33 months. However, we believe that if a patient has a high ischaemic risk, a patient should be treated with dual antiplatelet therapy for as long as the bleeding risk is acceptable; in some patients this could mean lifelong treatment. Since risk stratification is a dynamic process, cardiologists should perform periodical reassessment of a patient’s ischaemic and haemorrhagic risk. This requires routine, yearly outpatient visits including regular check-ups for haemoglobin, thrombocytopenia, and creatinine clearance, for as long as a patient is on DAPT. Finally, with regards to the ischaemic risk, DAPT and its duration are just one factor in the secondary prevention of cardiovascular events. Other important include lipid-lowering treatment, treatment of hypertension and diabetes, and other strategies for risk factor modification and lifestyle changes such as diet, exercise and smoking cessation.
